# Primary Tricuspid Regurgitation: From Neglect to Clinical Relevance

**DOI:** 10.3390/jpm15110535

**Published:** 2025-11-03

**Authors:** Mariagrazia Piscione, Jad Mroue, Dario Gaudio, Vivek Mehta, Fadi Matar

**Affiliations:** 1 Fondazione Policlinico Campus Bio-Medico, University of Rome, Via Alvaro del Portillo, 200, 00128 Rome, Italy; dario.gaudio@unicampus.it; 2 Cardiology, University of South Florida, Tampa, FL 33620, USA; jmroue1@usf.edu (J.M.); mcv@usf.edu (V.M.)

**Keywords:** primary tricuspid regurgitation, tricuspid valve surgery, transcatheter tricuspid valve repair, right heart disease

## Abstract

Primary tricuspid regurgitation (TR) is an underrecognized valve disease characterized by structural abnormalities of the tricuspid valve (TV) apparatus, including leaflet prolapse, flail, rheumatic degeneration, carcinoid involvement and congenital malformations such as Ebstein’s anomaly. Historically neglected and often misclassified as functional, primary TR has recently gained attention due to advances in multimodality imaging and increased awareness of its pathophysiological complexity and adverse outcomes. A major challenge that remains is the accurate diagnosis of primary TR, as well as the optimal timing for intervention, particularly in asymptomatic patients. While surgical repair or replacement has been the traditional approach, recent developments in transcatheter therapies, such as tricuspid edge-to-edge repair, have broadened the therapeutic landscape for patients considered at high surgical risk. In this context, personalized medicine has emerged as a central paradigm in the management of this valvular disease. Tailored therapeutic decisions should include anatomical, functional, and clinical parameters, as well as patient-specific risk factors such as age and comorbidities. Advanced imaging modalities, including 3D echocardiography and cardiac magnetic resonance, are essential for guiding this individualized approach. This review summarizes the current understanding of the etiology, pathophysiology, diagnostic tools, and treatment strategies for primary TR, highlighting the critical role of personalized treatment pathways in optimizing clinical outcomes.

## 1. Introduction

### 1.1. Epidemiology and Clinical Relevance of Tricuspid Regurgitation

Long forgotten in both clinical practice and research, tricuspid regurgitation (TR) is now receiving renewed attention as part of a broader interest in the right heart and its valvular apparatus [1]. TR affects approximately 1.6 million individuals, with 200,000 new cases diagnosed each year in the United States alone [2]. It was long regarded as a benign condition, until its prognostic implications were recognized [1].

### 1.2. Classification of Tricuspid Regurgitation

Secondary or functional TR accounts for 75% of cases, and it is typically due to pulmonary hypertension, right ventricle (RV) dilatation or dysfunction [3]. Primary or organic TR is less frequent (10–20%) and results from intrinsic defects of the tricuspid valve (TV) components: leaflets, annulus, chordae tendinae, and papillary muscles [3].

### 1.3. Anatomical Features of Tricuspid Valve and Pathophysiology of Primary Tricuspid Regurgitation

**Figure 1:**
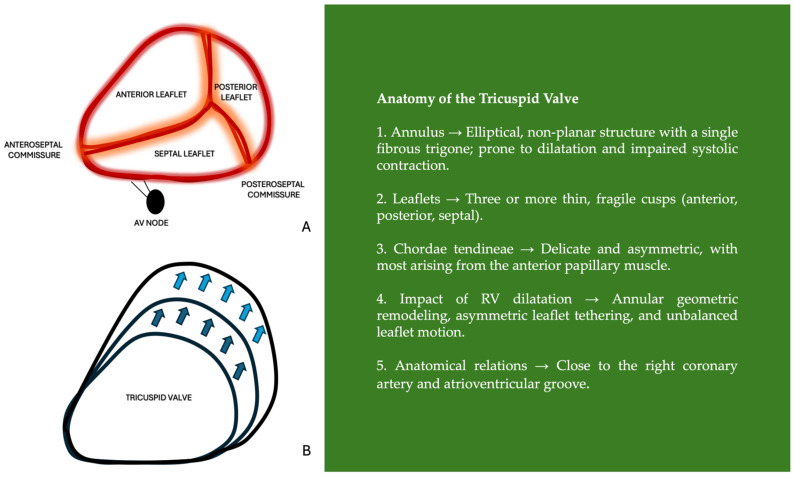
Anatomy of the tricuspid valve. (**A**) Representation of the tricuspid valve leaflets and commissures. (**B**) Annular enlargement. Abbreviations: RV: right ventricle.

The incidence of primary TR may be underestimated. Even cases with mixed or primary etiologies are often misclassified as secondary TR, as the intrinsic anatomy of the TV—with its thin leaflets, fragile chordae, and complex subvalvular apparatus—makes the valve prone to structural damage and degeneration of its components [3]. Unlike the dense, distinct fibrous structure that forms the mitral valve (MV) annulus, the TV annulus, which contains only a single fibrous trigone, merges with the fibro-fatty tissue of the right atrium (RA), making it more susceptible to dilation or inadequate systolic contraction [4]. Secondly, the three leaflets are supported by only two papillary muscles, with chordal attachments to the anterior leaflet arising predominantly from the anterior papillary muscle rather than from both. This asymmetry can contribute to structural instability and make the valve apparatus more vulnerable to mechanical stress [3]. Thirdly, while the septal leaflet is the smallest and relatively fixed, RV expansion can easily tether or restrict the anterior and posterior leaflets [3]. In the context of RV dilatation, geometric remodeling of the TV annulus and subvalvular apparatus can result in asymmetric leaflet tethering. In particular, tethering of one leaflet—most often the posterior or septal—may paradoxically lead to excessive motion or apparent billowing of the opposing leaflet, a phenomenon referred to as pseudoprolapse. This occurs because the restricted leaflet is pulled downward and outward by apical displacement of the RV wall, while the relatively unrestricted leaflet moves freely toward the RA during systole, mimicking true prolapse [5]. Differentiating true prolapse from pseudoprolapse is critical, as their underlying mechanisms and therapeutic implications differ significantly [3] (Figure 1).

### 1.4. Prognostic Implications of Severe Tricuspid Regurgitation

The prognostic implications of untreated TR have been increasingly recognized [3]. Severe TR, even in the absence of advanced left-sided disease, is independently associated with excess mortality, heart failure hospitalization, impaired exercise capacity, and reduced quality of life [4]. Adverse outcomes are largely mediated by progressive RA and RV remodeling, eventually culminating in right-sided heart failure and systemic venous congestion [4]. Recent advances in imaging and longitudinal cohort data have highlighted that earlier identification and intervention in TR may improve outcomes and prevent irreversible right heart damage [5].

### 1.5. Rationale and Aims of the Review

This review will focus on primary TR and its different etiologies indicating a broad range of clinical phenotypes, frequently presenting with nonspecific symptoms and advancing gradually [5,6]. Recent innovations in imaging technologies and an increasing volume of longitudinal data have highlighted the prognostic meaning of untreated TR [7], transforming the approach from late surgical intervention in advanced disease to early, individualized management strategies [6,7]. In this scenario, primary TR is no longer an incidental finding but a target for proactive evaluation and patient-tailored treatment. As part of the approach toward personalized cardiology, understanding the mechanisms, risk stratification tools, and therapeutic options for primary TR is essential to optimize outcomes and quality of life of affected patients.

## 2. Etiology, Pathophysiology and Treatment

### 2.1. Tricuspid Valve Prolapse

#### 2.1.1. TVP: Comparison with MVP and Knowledge Gaps

The pathogenesis of tricuspid valve prolapse (TVP) is largely unexplored, and it is still debated whether TVP represents a distinct valvular entity or simply a more advanced manifestation of valvular myxomatous degeneration, involving both left and right heart valves [8,9,10]. The mechanisms underlying mitral valve prolapse (MVP) have been more extensively studied. MVP is defined by thickening, elongation, and redundancy of the mitral leaflets, resulting from pathological remodeling of the valve structure [11]. This process is mediated by the activation of myofibroblast-like cells, which express smooth muscle-associated contractile proteins, matrix metalloproteinases, and inflammatory cytokines [11]. In turn, these factors promote extracellular matrix degradation and disorganization, leading to the expansion of the proteoglycan-rich spongiosa layer and to the degeneration of collagen and elastin fibers within the fibrosa and the atrialis layers [11]. As the disease progresses, morphological changes extend beyond the leaflets: the mitral annulus dilates and flattens, losing its physiologic saddle shape, and the *chordae tendineae* elongate or rupture, ultimately resulting in leaflet flail and severe MR [11,12].

So, while the etiology of MVP is well characterized, as discussed, TVP remains a poorly understood and underrecognized entity, with largely unknown pathophysiological mechanisms (Table 1). Moreover, its low prevalence and the technical limitations of transthoracic echocardiography (TTE) make TVP difficult to study in detail [9]. As a matter of fact, in total less than fifty cases of TVP have been described in the literature, raising questions about its true prevalence [9]. Consensus on specific diagnostic thresholds or definitions among physicians still lacks, further complicating the identification and the study of this entity [9] (Table 2).

#### 2.1.2. Evidence from Imaging Studies

To partially address this knowledge gap, the following two studies aim to estimate the incidence of TVP in the general population and assess whether TVP coexists with MVP, suggesting a possible spectrum of a shared valvular disorder.

Lorinsky et al. analyzed 218,943 TTE in eight years from a clinical database at Beth Israel Deaconess Medical Center. 410 patients (0.3%) had suspected TVP [9]. They demonstrated that an atrial displacement (AD) > 2 mm in the parasternal short axis view had the best accuracy against suspected TVP to identify TVP [9].

According to another study by Guta et al., considering 94 patients with TVP evaluated ed with cardiac magnetic resonance (CMR) among 263 with MVP, TVP incidence was estimated to be between 0.03% and 0.86% [10]. More specifically, Guta et al. used two CMR views of the RV to detect TVP: the 4-chamber view (septal and posterior leaflets) and the inflow-outflow view (anterior and posterior leaflets) [10]. The suggested CMR criteria for TVP specified a RA excursion of 3 mm for the septal leaflet and 2 mm for the anterior and posterior leaflets [8]. Nonetheless, it must be acknowledged that the assessment of the TV leaflets by CMR is more challenging than that of the mitral valve, due to the thinner structure of the TV leaflets and the wide anatomical variability in their configuration, which complicate accurate measurement and interpretation [10].

In addition, these studies have attempted to explore the pathophysiological mechanisms underlying TVP suggesting a potential connection in the etiology of MVP and TVP. Guta et al. reported that bileaflet MVP was significantly associated with the presence of TVP (47% vs. 24%), whereas no cases of TVP were identified among individuals without MVP [10]. Furthermore, TVP was more frequently encountered in patients with severe MR [10]. However, this association was not universally recognized [9]. These findings raise compelling questions about the etiology and classification of TVP [11]. Further histopathological and imaging-based investigations are needed to clarify whether TVP represents part of the spectrum of connective tissue valve diseases or a separate entity with unique anatomical and functional correlates [11,12].

#### 2.1.3. Current Surgical and Transcatheter Perspectives

Current European and American Guidelines recommend surgical indication for symptomatic severe primary TR without RV dysfunction [16,17]. The surgical management of severe TR focuses primarily on restoring proper leaflet coaptation and valve competence [16,17]. The cornerstone of most surgical repairs is annuloplasty, a procedure that aims to reduce and reshape the dilated TV annulus [13]. This can be achieved either by implanting a prosthetic ring, which offers durable support and maintains annular geometry, or through suture-based techniques, such as the De Vega annuloplasty, which is simpler and does not require prosthetic material, although it may be less durable in the long term [13]. In cases where annular reduction alone is insufficient to achieve leaflet closure, additional leaflet procedures are often employed. One commonly used technique is the Kay bicuspidalization, in which the posterior leaflet is sutured to the septal leaflet, effectively converting the valve from a three-leaflet (tricuspid) to a two-leaflet (bicuspid) configuration, improving coaptation and reducing TR [13]. Another advanced method is the “clover technique,” which involves suturing the free edges of all three tricuspid leaflets together at their midpoints, creating a central point of coaptation and a clover leaf-shaped orifice. [13]. These techniques, used alone or in combination depending on the anatomy and mechanism of TR, aim to preserve the native valve and avoid prosthetic replacement, which is generally associated with higher long-term complications, especially in young or high-risk patients [13]. In recent years, transcatheter TR repair has emerged as a viable therapeutic option and it is being increasingly adopted in clinical practice [18]. There is the possibility to use transcatheter tricuspid valve replacement (TTVR) but even though new devices have promising efficacy in reducing TR, their use raises concerns about their potential to cause high bleeding risk and interference with the cardiac conduction system [13]. The most recent European Society of Cardiology (ESC) guidelines provide a Class IIa recommendation for tricuspid trans edge-to-edge repair (T-TEER) in patients with symptomatic severe TR who are deemed inoperable, provided the procedure is performed in experienced heart valve centers [16,18]. The study by Dannenberg et al. focuses on T-TEER in patients with primary TR due to prolapse, with the key objectives to assess the feasibility, procedural success, and early post-intervention reduction in TR severity in this specific subgroup compared to the functional TR group [14]. According to the results, T-TEER provides a similarly safe and effective reduction in TR in both forms actually a decrease to TR of more than two degrees was achieved in 76% of primary TR and 78% of secondary TR. [13]. Another study by Sugiura et al. demonstrated the efficacy of Triclip^®^ (Abbott, Santa Clara, CA, USA) implantation in patients with primary TR [15]. These findings broaden the therapeutic role of transcatheter therapies in the management of degenerative tricuspid valve disease [15]. However, it should be emphasized that the current evidence supporting T-TEER in primary TR, including cases due to leaflet prolapse, is derived almost exclusively from observational cohorts, with no randomized controlled trials available to date. Larger prospective studies are needed to confirm safety, durability, and long-term outcomes.

### 2.2. Tricuspid Valve Flail

#### 2.2.1. Definition and Clinical Significance

Flail tricuspid leaflet is a prolapsed segment of the TV with systolic displacement of the leaflet tip and/or ruptured *chordae* into the RA [1]. This condition leads to a form of organic, severe, and typically irreversible TR.

#### 2.2.2. Etiology and Clinical Presentation

In a multicenter analysis, Messika-Zeitoun et al. investigated the etiology, clinical presentation, and outcomes of 60 patients with flail of TV leaflets [19]. The majority (62%) had a traumatic origin, with half resulting from blunt chest trauma—most commonly due to motor vehicle accidents—and the other half was from iatrogenic causes, particularly chordal rupture during RV endomyocardial biopsy (EMB) [19]. Among non-traumatic cases, myxomatous degeneration (12%), infective endocarditis (8%), and congenital anomalies (3%) were identified, while 15% of cases were classified as idiopathic due to the absence of a defined cause [19]. The link between trauma or iatrogenic injury and flail of TV leaflet has long been recognized. As early as 1958, Parmley et al. reported TV papillary muscle rupture following non-penetrating chest trauma [20], and multiple subsequent series have described similar cases [21]. Additionally, EMB, especially in post-heart transplant patients, has been implicated in causing iatrogenic flails [15]. Studies have reported a prevalence of flail of TV leaflets ranging from 2.3% to 25%, with moderate-to-severe TR observed in up to 85% of patients post-EMB, highlighting the clinical relevance of procedure-related TR in transplant populations [22,23].

Another recognized cause of TV leaflet flail is related to lead extraction, which can result in mechanical damage of the subvalvular apparatus or leaflet tearing during device removal [22]. A recent study suggested that TR resulting from lead extraction was strongly associated with the use of additional extraction tools beyond simple traction, and with female sex—possibly due to a more pronounced fibrotic response and/or more fragile valvular and subvalvular structures in women [24].

#### 2.2.3. Therapeutic Implications

Given the potentially severe consequences of TR caused by flail leaflets, early surgical intervention should be considered in symptomatic patients before the development of unresponsiveness to treatment and right heart failure [25]. In asymptomatic individuals, severe enlargement of the RA or RV on baseline TTE is a strong predictor of adverse outcomes, and such patients should be closely monitored and evaluated for timely surgical referral [21,22].

#### 2.2.4. Surgical and Transcatheter Options

Surgical TV repair remains the preferred strategy when anatomically feasible. This includes ring annuloplasty, edge-to-edge techniques (e.g., the clover technique), and chordal reconstruction using expanded polytetrafluoroethylene (ePTFE) neochordae [22]. However, when leaflet destruction is extensive or flail involves multiple segments with poor residual support, valve replacement becomes necessary. In such cases, bioprosthetic valves are favored, particularly in younger patients and those at risk for poor anticoagulation compliance [22]. About T-TEER, small observational studies and registry data, including subgroups from the TRILUMINATE study, report successful reduction in TR severity and improvement in functional status in selected patients with leaflet flail [18]. However, the long-term durability and optimal patient selection criteria for T-TEER in flail TV disease remain to be fully established, and surgical repair is often preferred when anatomy allows [18].

### 2.3. Rheumatic Disease

#### 2.3.1. General Features

Rheumatic heart disease is characterized by valvulitis, which typically damages the mitral valve, causing MR [26]. Isolated right-sided cardiac valve involvement, particularly TV, without any associated MV illness that leads to RV failure is an extremely rare finding, particularly during screening [26]. Rheumatic pathology typically leads to leaflet thickening, fibrosis, and retraction, often accompanied by commissural fusion. Inflammatory involvement may also extend to the chordae tendineae, further contributing to leaflet retraction [26,27]. These changes result in combined TV stenosis and regurgitation, with regurgitation usually being the predominant lesion [26,27].

#### 2.3.2. Epidemiology and Surgical Context

While the incidence of rheumatic TV disease has declined in developed countries, it still features prominently in both historical and contemporary surgical series, particularly in patients undergoing isolated TV surgery [24,28]. Notably, the vast majority of these patients had previously undergone MV surgery, reflecting the downstream impact of rheumatic involvement in multivalvular disease.

#### 2.3.3. Clinical Context and Diverging Viewpoint

This condition raises an important clinical debate regarding the optimal management of the TV during left-sided valve procedures, particularly in the context of mitral stenosis [26]. While current guidelines recommend TV repair in patients with severe TR undergoing left-sided surgery (Class I), the indications for intervening in mild or moderate TR remain controversial (Class IIa if moderate TR, Class IIb in the presence of mild TR and annular dilation) [16,27]. Some authors advocate for a liberal use of TV annuloplasty regardless of TR severity, citing its low procedural risk, reproducibility, and potential to prevent progressive right-sided dysfunction [29,30,31,32]. Others support a more conservative approach, arguing that mild TR may regress following correction of the left-sided defects, and that unnecessary intervention may even result in iatrogenic tricuspid stenosis—particularly when the left-sided procedure involves mitral or aortic stenosis, as the rheumatic disease often affect multiple valves simultaneously [31,32]. Recent evidence, including the study by Kim et al., indicates no clear clinical benefit from prophylactic TV repair during rheumatic mitral surgery in the absence of annular dilation, highlighting the need for individualized treatment decisions—especially in the presence of atrial fibrillation and RA enlargement [33].

### 2.4. Endocarditis

#### 2.4.1. Epidemiology and Risk Factors

Right-sided infective endocarditis (RSIE) accounts for approximately 5–10% of all cases of infective endocarditis and involves the TV in the vast majority of cases [34]. Actually, isolated pulmonary valve endocarditis is exceptionally rare, and sporadic involvement of structures such as the Eustachian valve, the interventricular septum, or the RV free wall has also been documented [35,36]. The epidemiology of RSIE significantly differs between industrialized and developing countries. In high-income settings, intravenous drug use represents the most common predisposing factor and it involves the TV in up to 90% of cases [37]. Conversely, in resource-limited settings, septic abortion and intra-abdominal sepsis remain significant contributors [38]. The minority of cases of RSIE is predominantly linked to the presence of intracardiac devices such as central venous catheters, pacemaker leads, implantable defibrillators, and prosthetic TV [39]. Hemodialysis patients are particularly vulnerable due to repeated vascular access and the presence of tunneled catheters, which facilitate direct bacterial entry into the right heart chambers [39,40]. Despite these risks, TV involvement in hemodialysis-associated RSIE remains relatively uncommon compared to left-sided forms, highlighting the selective pathogenetic mechanisms involved [41]. As device utilization increases globally, a shift in the RSIE landscape toward healthcare-associated etiologies is emerging, warranting heightened surveillance and tailored preventive strategies.

#### 2.4.2. Microbiological Profile

*Staphylococcus aureus* represents the predominant etiologic agent of RSIE, both in intravenous drug users (IDUs) and in non-addicted patients, accounting for the majority of cases [42]. Other frequently identified pathogens include *coagulase-negative staphylococci* and various streptococcal species [43]. Less common organisms implicated in RSIE include *Lactobacillus* spp., members of the HACEK group (*Haemophilus aphrophilus*, *Actinobacillus actinomycetemcomitans*, *Cardiobacterium hominis*, *Eikenella corrodens*, and *Kingella kingae*), *Enterococci*, and *Fungi* [43]. Fungal endocarditis is a rare entity overall and it is typically caused by *Candida* spp., and more rarely *Aspergillus* spp., particularly in immunocompromised hosts such as those with Human Immunodeficiency Virus (HIV) infection [43]. Although the microbiologic profile of RSIE largely overlaps with that of left-sided infective endocarditis (LSIE), its pathophysiological mechanisms may differ. Actually, direct inoculation of pathogens into the venous circulation is believed to facilitate the preferential localization of infection to the right heart. However, the higher incidence of RSIE in this population is also attributed to unique immunologic responses, endothelial injury, and the nature of the injected substances and contaminants, which may enhance pathogen adherence to the TV [44,45]. These factors, in combination with the intrinsic hemodynamic and anatomical features of the right heart, contribute to the complex pathogenesis of the disease [45].

#### 2.4.3. Diagnostic Imaging

Unlike LSIE, TTE is often sufficient to establish the diagnosis of RSIE, especially in IDUs, where large TV vegetations are frequently well-visualized [45]. Some studies have reported that TEE does not significantly improve diagnostic accuracy over TTE for detecting TV vegetations in this population [46]. Nonetheless, TEE remains valuable in selected cases, particularly for evaluating vegetations on pulmonary valves, prosthetic devices, central venous catheters, and implantable cardioverter-defibrillators, or when RSIE arises from atypical locations. TEE is also superior in identifying complications like perivalvular abscesses or when clinical response to therapy is suboptimal [47].

#### 2.4.4. Medical Management

Empirical antimicrobial therapy for suspected RSIE should be initiated promptly after obtaining adequate blood cultures, and must be directed toward the most likely causative pathogens, actually empirical treatment typically includes an anti-staphylococcal agent with activity against *methicillin-resistant Staphylococcus aureus* (MRSA). Recommended regimens include the combination of vancomycin and gentamicin, or daptomycin as monotherapy [48]. Once the causative organism and its antibiotic susceptibility profile have been identified, antimicrobial therapy should be adjusted accordingly [49]. The total duration of treatment usually depends on pathogen type, response to therapy, and clinical evolution [50]. In most of cases (70–80%) RSIE can be successfully managed with conservative antimicrobial therapy, while the remaining patients require surgical intervention [51].

#### 2.4.5. Indication for Surgery and Surgical Strategies

**Figure 2:**
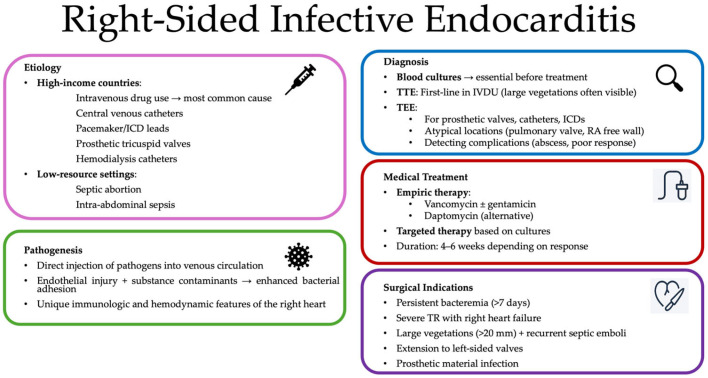
Overview of the clinical approach to right-sided infective endocarditis. Abbreviations: ICD: implantable cardioverter defibrillator, TEE: trans-esophageal echocardiogram, TTE: trans-thoracic echocardiogram, RA: right atrium, TR: tricuspid regurgitation.

The commonly accepted indications for surgical intervention in RSIE—in patients who are already receiving appropriate antimicrobial therapy—include several high-risk clinical scenarios. These are largely based on observational studies and expert consensus and can be summarized as follows: persistent bacteraemia lasting more than one week despite adequate and targeted antibiotic treatment; RV dysfunction due to acute severe TR; respiratory failure necessitating mechanical ventilatory support, typically secondary to recurrent septic pulmonary *emboli*; extension of infection to left-sided cardiac structures; and presence of large residual tricuspid vegetations (>20 mm) in the context of recurrent pulmonary embolization [48]. Surgical management of TV infective endocarditis (IE) includes three strategies: valve repair, valve replacement, and, more rarely, surgical valvectomy [45]. Although the overall recurrence rate of IE remains low, the durability of TV repair becomes particularly relevant in the population of IDUs, who are at heightened risk for relapse, reinfection, and the need for reoperation [48,49,50,51,52,53,54] [Figure 2]. In this context, surgical strategy selection is critical, and multiple repair techniques have been described in the literature [54]. These include ring or suture-based annuloplasty, the Kay bicuspidalization technique, and in selected cases, valvectomy without valve replacement, particularly when concern exists regarding reinfection of prosthetic material [53,54]. In general, TV repair is preferred in cases of right-sided IE due to its association with better short- and long-term outcomes, particularly in terms of lower recurrence rates of infection and reduced need for reintervention [54]. According to a recent study, from January 2002 to January 2015, 134 adults with RSIE were enrolled and underwent surgery [50]. Patients were stratified based on underlying predisposing conditions and subsequent analyses focused on in-hospital outcomes, long-term mortality, and the incidence of reoperation due to recurrent IE over time [50,55]. Their surgical experience emphasized the importance of prioritizing TV repair over replacement whenever feasible. In particular, the authors describe repair techniques involving leaflet reconstruction with autologous pericardial patches or synthetic materials, combined with artificial chordae to restore mobility and coaptation [50,55]. Despite optimized surgical techniques, a significant number of patients leave the operating room or hospital with residual moderate or even severe TR, highlighting the technical complexity of achieving durable repair in advanced disease [50,55]. In extreme cases, valvectomy without valve replacement may be performed, particularly in IVDUs [50,55]. However, this strategy is only advisable when pulmonary vascular resistance is low, as patients with elevated pulmonary hypethension are unlikely to tolerate the resulting severe TR. In general, valvectomy is considered a bridge strategy, with delayed valve replacement required after infection resolution [55]. Moreover, in patients with combined RSIE and invasive left-sided (aortic and mitral) endocarditis, the prognosis is significantly worse [55]. These complex cases often necessitate radical surgical reconstruction, including replacement of both valves and reconstruction of the intervalvular fibrosa, typically using the “commando” or “hemi-commando” procedures, which remain high-risk but potentially life-saving interventions [55]. Nonetheless, when repair is not possible and valve replacement becomes necessary, also in this occasion, bioprostheses are favored over mechanical prostheses [55]. Intraoperative considerations also include the prophylactic placement of permanent epicardial leads, particularly in patients with intraoperative heart block, to prevent future damage to a replaced valve during potential transvenous lead insertion and to minimize the risk of reinfection [55].

#### 2.4.6. Novel Approaches

More recently, attention has been drawn to novel percutaneous techniques aimed at the aspiration of large vegetations using extracorporeal circuits [56]. Following the 2014 approval by the U.S. Food and Drug Administration (FDA) for the percutaneous removal of intravascular debris, the AngioVac^®^ system has gained increasing attention as a minimally invasive alternative to surgical intervention—particularly in patients deemed high-risk surgical candidates [56]. This transcatheter aspiration device enables the removal of blood and undesired intravascular material via an extracorporeal circuit incorporating an inline filter, before reinfusion of the filtered blood into the patient. This approach facilitates the debulking of large or persistent vegetations, thereby reducing bacterial load, enhancing the efficacy of antimicrobial therapy, and potentially lowering the risk of recurrence [57]. Its clinical utility was further demonstrated in a study by Schaerf et al., which evaluated 20 high-risk patients with cardiac device-related IE and vegetations refractory to medical treatment [57]. Additionally, a recent systematic review and meta-analysis including 301 patients reported favorable outcomes associated with AngioVac^®^-assisted aspiration, highlighting effective removal of right-sided vegetations, clearance of bacteremia, and a relatively low rate of procedural complications in this high-risk cohort [58]. While these results underscore the potential of AngioVac^®^ in managing TV or pulmonary valve vegetations, evidence supporting its use in other contexts, such as endovascular vegetation extraction, remains limited.

### 2.5. Carcinoid Heart Disease

#### 2.5.1. Pathogenesis and Right-Sided Involvement

Carcinoid heart disease (CHD) is caused by circulating vasoactive substances, such as prostaglandin, histamine, bradykinin, serotonin or 5-hydroxytryptamine, tachykinins (substance P, neurokinin A, neuropeptide K), and transforming growth factor-β released by neuroendocrine tumors (NTs), most commonly originating in the gastrointestinal tract [59]. Among CHD manifestations, valvular heart disease, particularly involving the right-sided valves, represents the hallmark and most severe cardiac complication of this condition [60]. Actually, when NTs metastasize to the liver, the substances bypass hepatic degradation and enter the systemic circulation via the hepatic veins through the inferior vena cava reaching the RA. The lungs metabolize and inactivate many of the vasoactive chemical peptides, particularly serotonin, via monoamine oxidase in pulmonary endothelial cells [60]. Therefore, left-sided heart involvement is rare, unless there is a right-to-left shunt (e.g., patent foramen ovale) or the tumor is in the lungs or the left heart is directly exposed to carcinoid secretions [60]. Nonetheless, when valvular involvement occurs, it often has a greater impact on prognosis than the tumor burden itself [60].

#### 2.5.2. Clinical Implications and Imaging Evaluation

Given its prognostic significance, CHD, cardiologists should actively investigate potential cardiac involvement whenever a NTs is diagnosed, particularly in the presence of hepatic metastases or elevated levels of serotonin and its metabolites [61]. In this context, cardiac imaging—including TTE and, increasingly, advanced modalities such as CMR and cardiac computed tomography—plays a central role in both the initial assessment and the longitudinal management of affected patients [61]. In TTE evaluation of TR, the hallmark morphological pattern is a dilated annulus and diffusely thickened, fibrotic, and retracted leaflets [62]. These leaflets exhibit markedly restricted mobility, failing to coapt in systole—thus leaving a large central regurgitant orifice—and remaining partially fixed in diastole. Loss of leaflet pliability is a key diagnostic clue, and typically, all three leaflets are involved [62]. Additionally, chordae tendineae may appear thickened and shortened, and papillary muscles may occasionally be affected. The use of 3DTTE can enhance assessment of both leaflet and subvalvular abnormalities, improving diagnostic accuracy [62,63]. From a functional standpoint, at least severe TR frequently results in a hemodynamic scenario where the RA and RV function almost as a single chamber. In contrast, tricuspid stenosis is rare in CHD [63]. Doppler evaluation typically shows a low mean transvalvular gradient (<5 mmHg), largely due to increased flow volume rather than stenotic physiology [61]. The regurgitant jet on continuous-wave Doppler is often “dagger-shaped”, reflecting early systolic peaking and rapid decline of the pressure gradient due to elevated RA pressure. Despite the severity of TR, peak regurgitant velocity is usually <2 m/s, consistent with normal or mildly elevated RV pressures [64,65].

#### 2.5.3. Surgical Management

Since, as already said, TR is associated with higher mortality than metastatic progression alone, surgical intervention—most commonly TV replacement—is recommended in symptomatic patients with right heart failure, progressive right-sided chamber dilatation, or prior to hepatic resection, unless contraindications exist [66]. The surgical literature documents multiple series of patients with CHD undergoing isolated or combined TV surgery, with the most comprehensive data derived from the Mayo Clinic’s 30-year experience [60]. Actually, Nguyen et al. demonstrated a significant improvement in perioperative outcomes over time, with early mortality declining from 29% (1985–1994) to 7% (1995–2004) and further decreasing to 5% in more recent years, likely reflecting better patient selection, surgical techniques, and perioperative care [66]. Although surgical valve replacement has been the traditional therapeutic standard, many patients with CHD are deemed high-risk surgical candidates due to advanced disease, liver involvement, or general frailty [53].

#### 2.5.4. Transcatheter Therapies

Transcatheter therapies have gained increasing attention in this context. T-TEER has shown promise in functional TR but pose significant limitations in CHD. The fibrotic and retracted nature of TV leaflets in CHD frequently prevents adequate grasping and coaptation [18]. Recent case series have explored TTVR as an alternative strategy. Devices like the Evoque valve (Edwards Lifescience) have been evaluated in early feasibility studies, including patients with organic TR [67]. Although none of these trials specifically enrolled CHD patients, isolated reports suggest that TTVR may overcome the anatomical challenges of fibrotic leaflet retraction and provide more durable TR reduction in this population [67,68]. Despite these promising advances, data on transcatheter treatment in CHD remain limited, with only anecdotal evidence or small series reported. Larger prospective studies and registries are needed to evaluate feasibility, safety, and long-term outcomes of transcatheter therapies in patients with CHD-related TR [68]. The management of TR in CHD represents a paradigmatic example of personalized therapy in clinical practice. Although TR is often torrential in this setting, its pathogenesis is distinct—more fibrotic and restrictive than atrial functional or degenerative—and shares several features with rheumatic valve disease [69]. Therefore, therapeutic strategies must be carefully tailored to the underlying pathology and the overall clinical status of the patient. In addition to surgical or interventional approaches, medical therapy with somatostatin analogs plays a pivotal role in preventing further cardiac involvement by inhibiting the systemic spread of serotonin and other vasoactive substances released by NTs [70] (Table 3).

### 2.6. Ebstein’s Anomaly

#### 2.6.1. Epidemiology and Clinical Presentation

Congenital TR includes a wide spectrum of different pathologies, including Ebstein’s anomaly (EA), tricuspid atresia, and cleft of the TV typically associated with atrioventricular septal defects [71]. Among these, EA is the most frequently encountered in clinical practice, often presenting for the first time during adulthood associated with supraventricular arrhythmias—particularly atrial fibrillation or flutter—due to chronic RA dilation. However, the development of biventricular failure and ventricular arrhythmias portends a worse prognosis and is associated with significantly reduced survival [71]. More in detail, EA is a rare congenital malformation (1: 20,000) characterized by TV dysplasia and regurgitation, often associated with RV myopathy and dysfunction [71].

#### 2.6.2. Embryology and Pathogenesis

First described by Wilhelm Ebstein in 1866, significant advances have been made in the understanding, diagnosis, and management of this complex disease [72]. The condition originates from incomplete delamination of the septal leaflet of the TV from the interventricular septum during embryogenesis, resulting in apical displacement and impaired valve function [73].

#### 2.6.3. Key Anatomical Features

Key features of the disease include: failure of delamination of the TV leaflets, particularly the septal leaflet; apical displacement of the functional tricuspid orifice, leading to a downward migration of the septal leaflet into the right ventricle; dilatation and partial “atrialization” of the RV due to the enlarged RA staying on ventricular territory; abnormalities of the anterior leaflet, such as fenestrations, tethering, or redundancy; and dilatation of the right atrioventricular junction [74]. The most distinctive anatomical criterion differentiating EA from other congenital tricuspid lesions is the degree of AD of the septal leaflet, which is considered diagnostic when it measures ≥8 mm/m^2^ of body surface area [74]. Modern imaging modalities, including three-dimensional TEE and CMR have substantially enhanced our ability to assess the anatomical and functional abnormalities in detail [75,76].

#### 2.6.4. Surgical Management and Outcomes

Parallel to these diagnostic improvements, surgical techniques have also evolved, with the cone reconstruction procedure now considered the standard approach in many centers. This technique reconfigures the malformed valve leaflets into a more functional, conical structure, significantly improving valve competence [75,76,77]. As a result, surgical outcomes have dramatically improved: whereas historical operative mortality approached 50%, current data suggest a 20-year survival exceeding 70% following TV repair or replacement in adult patients [75,76,77]. In a recent contribution to the Journal of the American College of Cardiology, Egbe et al. sought to identify the most prognostically relevant TTE parameters of RV function in patients with EA [76]. Utilizing data from 620 adults enrolled in the Mayo Clinic Adult Congenital Heart Disease registry, the study compared four commonly used indices of RV systolic function: RV global longitudinal strain (RVGLS), RV fractional area change, RV s′ velocity, and tricuspid annular plane systolic excursion [76]. Among these, RVGLS emerged as the strongest predictor of mortality, underscoring the notion that EA functions primarily as a right ventricular myopathy, with progressive RV dysfunction driving long-term cardiovascular deterioration [76,77]. Timely surgical repair is therefore a cornerstone in the management of EA, as delayed intervention is associated with poorer long-term outcomes.

### 2.7. Radiation-Induced Tricuspid Regurgitation

#### 2.7.1. Epidemiology and Clinical Relevance

Radiation-induced valvular heart disease (VHD) is a recognized late complication of mediastinal radiotherapy, particularly in patients treated for lymphoma, breast cancer, or other thoracic malignancies (especially lung and esophageal cancers) [78]. Notably, among patients cured for Hodgkin lymphoma, cardiovascular complications represent the second most common cause of treatment-related morbidity [79]. Several retrospective studies have investigated the prevalence of VHD in long-term survivors, albeit at different time intervals following radiation therapy [80].

#### 2.7.2. Prevalence and Valve Involvement

Valvular regurgitation is more frequently observed than stenosis, with the aortic valve being the most commonly affected site in cases of radiation-induced stenosis [81]. In a review by Heidenreich et al., the prevalence of at least mild VHD 10 years post-radiation was reported as follows: aortic regurgitation (AR) 5%, MR 26%, TR 9%, and pulmonic regurgitation (PR) 2% [82]. However, beyond 20 years from radiation exposure, the prevalence substantially increased: AR 60%, MR 52%, TR 26%, PR 12%, and aortic stenosis (AS) 16% [82]. The odds of developing AS increased nearly 14-fold for each additional decade after radiation therapy, even after adjusting for age and sex [82]. Despite the anterior anatomical location of the right-sided cardiac valves, most studies consistently report a higher prevalence of left-sided VHD, suggesting that factors beyond local radiation dose—such as differential hemodynamic stress or tissue susceptibility—may contribute to the development of radiation valvulopathy [83]. Importantly, the time elapsed since radiation exposure remains a critical determinant of disease severity, highlighting the need for long-term surveillance in this population [83]. Actually, radiation-induced TR is characterized by a progressive degenerative process that often begins with early leaflet retraction, leading initially to valvular regurgitation and, over time, to valve thickening and calcification, resulting in stenosis [83].

#### 2.7.3. Pathophysiology

The proposed mechanisms underlying radiation-induced valve calcification involve the activation of valvular interstitial cells (VICs) and their differentiation into osteoblast-like phenotypes, which actively promote calcium deposition within the valve interstitium [84]. This pathological remodeling is thought to be driven by specific cellular and extracellular matrix (ECM) responses to ionizing radiation [84]. In particular, activation of stromal fibroblasts and increased expression of transforming growth factor-β (TGF-β) contribute to excessive collagen deposition [84]. An in vitro study recently demonstrated that upregulation of matrix metalloproteinases (MMPs) by VICs leads to ECM remodeling, which plays a critical role in the progression of radiation-induced valvulopathy [84]. The balance between collagen synthesis and degradation is alterd, favoring ECM remodeling and progressive leaflet thickening [85]. Microcalcifications accumulate over time, eventually coalescing into macroscopic annular and leaflet calcification, which accounts for the combined fibrotic and stenotic phenotype observed decades after radiation exposure [85]. In many cases, the disease process extends beyond the valve leaflets to involve adjacent cardiac structures, including the annulus and the subvalvular apparatus [85,86].

#### 2.7.4. Clinical Practice and Imaging

**Figure 3:**
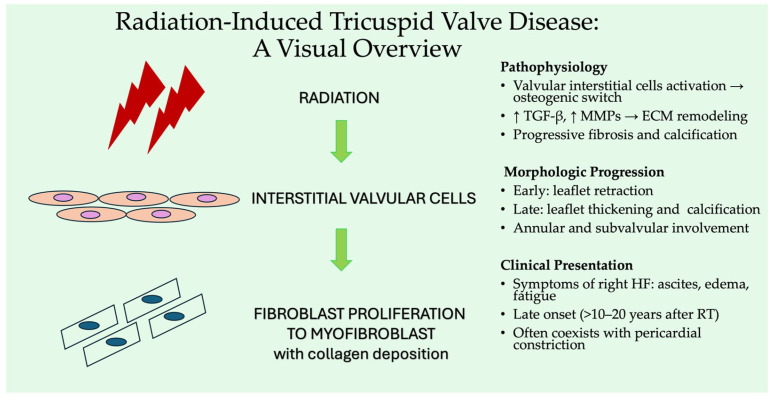
Pathophysiological cascade of radiation-induced tricuspid valve degeneration mediated by valvular interstitial cells. Abbreviations: ECM: extracellular matrix, HF: heart failure, MMPs: metalloproteinases, RT: radiotherapy, TGF-β: transforming growth factor β.

Clinically, radiation-related TR may manifest years to decades after radiation exposure and can be accompanied by other radiation-induced cardiovascular injuries, including pericardial constriction, coronary artery disease, and myocardial fibrosis [87]. Diagnosis is typically made by TTE or TEE, which may reveal thickened and retracted TV leaflets with poor coaptation and normal right ventricular geometry [82] (Figure 3).

#### 2.7.5. Management Challenges

Management of radiation-induced TR is challenging, especially in patients with multiple comorbidities or prior thoracic surgeries. Surgical intervention, when indicated, often carries increased risk due to tissue fragility and adhesions [88]. Emerging transcatheter therapies may offer a less invasive alternative in selected high-risk patients, though data are still limited in this subgroup [78]. Truong et al. described a paradigmatic case of a 67-year-old woman, previously treated for left breast angiosarcoma at age 30 with doxorubicin, cyclophosphamide, and high-dose chest radiation (∼120 Gy), presented with a 1-year history of worsening dyspnea, orthopnea, abdominal distension, and weight loss [78]. Her post-radiation course had been complicated by osteoradionecrosis of the chest and recurrent pleural effusions, requiring multiple thoracic surgeries. She had known TR on prior TTE. She was urgently admitted in shock, with altered mental status, hypotension, hypothermia, and renal failure, requiring venoarterial extracorporeal membrane oxygentaor (ECMO) support. Given her poor clinical status and lack of a functional sternum, she was deemed ineligible for surgical tricuspid valve intervention or advanced heart failure therapies [78]. Off-label T-TEER with MitraClip was considered. However, the optimal timing of TEER was debated due to concerns that TR reduction might have impaired RV function (by increasing afterload) or may have caused LV overload (via increased preload), potentially hindering ECMO weaning. Nonetheless, following T-TEER, the patient showed improved TR and was discharged in New York Heart Associated functional Class II; despite imaging signs of residual pericardial constriction, she remained clinically stable on diuretics with no heart failure hospitalizations after 5 years [78]. This case exemplifies the potential role of transcatheter therapies in radiation-induced TR, although the overall evidence base remains scarce and restricted to case reports or small series. No randomized data exist, and further studies are warranted to establish safety and efficacy in this complex patient population.

### 2.8. Sarcoid-Related Tricuspid Regurgitation

#### 2.8.1. Infiltrative Disease and Tricuspid Regurgitation

Although uncommon, infiltrative diseases such as sarcoidosis and amyloidosis can contribute to or cause TR, either through direct valvular infiltration or via myocardial involvement of the right heart [89].

#### 2.8.2. Cardiac Sarcoidosis: Pathophysiology and Mechanisms of Tricuspid Regurgitation

Sarcoidosis is a multisystem granulomatous disorder caused by a complex interplay of environmental, genetic, and immune-mediated factors, leading to the formation of noncaseating granulomas in various organs, including the lungs, eyes, skin, lymph nodes, and heart. Cardiac sarcoidosis (CS) refers to granulomatous infiltration of the myocardium, either as part of systemic sarcoidosis or, less commonly, in isolation [90]. Based on imaging and autopsy studies, up to 25% of patients with systemic sarcoidosis show evidence of cardiac involvement, although clinically manifest disease is less frequent [91]. Although rare, TR may occur in CS as a primary valve defect or most often secondary to RV and annular dilation resulting from myocardial involvement [89]. However, direct granulomatous infiltration of the TV has been documented, including a case in which histopathological examination following cardiac transplantation revealed non-caseating granulomas within the valve leaflets, moderator band, RV septum, and RA [92]. In that patient, extensive valvular fibrosis caused adherence of the TV leaflets to the RV wall, resulting in severe TR [91]. More commonly, RV inflammation leads to chamber dilation, with consequent annular enlargement and leaflet malcoaptation [91]. Although this mechanism reflects functional TR, concomitant inflammation of valve leaflets or papillary muscles may also contribute [91,92].

#### 2.8.3. Management of Cardiac Sarcoidosis-Related Tricuspid Regurgitation

Treatment of CS relies primarily on immunosuppressive therapy, typically corticosteroids and, in selected cases, additional immunomodulatory agents, aiming to control systemic inflammation and potentially reverse myocardial and valvular involvement [90]. However, in advanced stages with irreversible structural damage, management should be tailored to the severity of valvular dysfunction, with the therapeutic approach—be it medical, surgical, or transcatheter—being individualized, as current literature lacks robust data supporting one strategy over another [91,92].

### 2.9. Amyloid-Related Tricuspid Regurgitation

#### 2.9.1. Cardiac Amyloidosis: Pathophysiology and Prevalence of TR

While the understanding and treatment of CS remain limited, the landscape is markedly different for cardiac amyloidosis (CA). The recent advent of disease-modifying therapies has sparked significant interest in this field, leading to a deeper exploration of the pathophysiological mechanisms and a growing emphasis on early and accurate diagnosis [93]. This shift has substantially improved disease awareness and fostered the development of novel diagnostic tools, thereby enhancing clinical recognition and management of the condition even if CA involves valve pathology [93].

Moderate-to-severe TR is a common finding in patients with CA, with an estimated prevalence of up to 26% [94]. In the study by Tomasoni et al., isolated TR was observed in 12.3% of patients, while 22.3% had both MR and TR [93]. At the molecular level, amyloid fibril deposition within the valvular interstitium disrupts the layered architecture of the tricuspid leaflets, leading to thickening, loss of elasticity, and restricted motion. This infiltration is associated with increased stiffness of the extracellular matrix and impaired leaflet coaptation. In addition, amyloid deposits extend to the chordae tendineae and papillary muscles, further reducing subvalvular flexibility [95].

**Figure 4:**
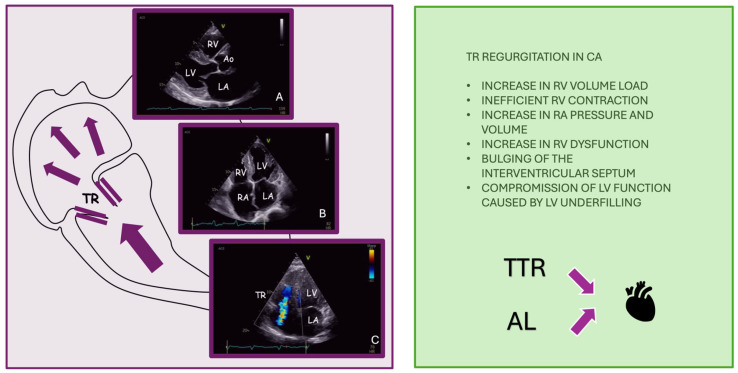
(**A**) the trans-thoracic echocardiogram (parasternal long axis view) demonstrates concentric myocardial hypertrophy and right ventricle free wall thickening (indirect sign of pulmonary hypertension). (**B**) the trans-thoracic echocardiogram (apical 4 chamber view) shows the sparkling, granular appearance of the myocardium. (**C**) the trans-thoracic echocardiogram (apical 4 chamber view) represents moderate tricuspid regurgitation by color Doppler assessing. Abbreviations: LV: left ventricle, RA: right atrium, RV: right ventricle, TR: tricuspid regurgitation, Ao: aortic valve, LA: left atrium, TTR: transthyretin, AL: amyloidosis AL.

The macroscopic underlying mechanism of TR in CA is variable: while in some cases it is related to RV dilation and pressure overload, in others it may be due to direct amyloid infiltration of the TV leaflets, resulting in thickening and restricted motion [95]. In CA, valvular defects result not only from amyloid infiltration of the leaflets and the subvalvular structures, but are also amplified by functional consequences such as restrictive ventricular filling and chronically elevated atrial and ventricular pressures, which together impair valve coaptation and promote regurgitation [95]. Severe TR leads to chronic volume overload of the right heart, initially compensated by the RV, but progressively resulting in RV dysfunction, septal shift, and reduced LV filling (Figure 4). These hemodynamic alterations are particularly detrimental in CA, where the myocardium is already stiff and poorly compliant [96].

#### 2.9.2. Therapeutic Management of CA-Related Tricuspid Regurgitation

Diuretics are the first-line therapy to manage congestion, but patients with CA often have low blood pressure and limited diuretic tolerance [97]. Surgical correction of TR is rarely performed due to the high operative risk, especially in elderly and frail patients [98]. In selected cases with favorable anatomy, T-TEER can be considered. Recent small case series, such as that by Hoerbrand et al., have shown that TEER is feasible and may improve symptoms, although procedural durability appears lower in CA than in other forms of functional TR [99]. Structural remodeling after TR correction also seems limited in CA, likely due to the underlying restrictive cardiomyopathy. Overall, TR in CA is frequent, multifactorial, and clinically relevant. Management remains challenging and should be individualized, balancing anatomical feasibility, hemodynamic burden, and overall clinical frailty [99].

## 3. Differential Diagnosis Between CA-Related and CS-Related TR

Differentiating CS from CA in the advanced forms can be challenging, as both conditions may present with biventricular myocardial thickening and granular speckling on echocardiography [84]. However, key distinguishing features include RV free wall thinning or aneurysms in CS versus concentric hypertrophy and valvular or interatrial septal thickening in CA. On CMR, both diseases show patchy areas of late gadolinium enchancement (LGE), but global subendocardial LGE is typical of CA, often with increased extracellular volume and elevated native T1 times (>1164 ms) [82]. In contrast, increased fluorodeoxyglucose (FDG) uptake on Positron emission Tomography (PET), particularly in the RV free wall, favors CS. When imaging is inconclusive, endomyocardial biopsy remains essential for definitive diagnosis [82].

## 4. Conclusions

**Figure 5:**
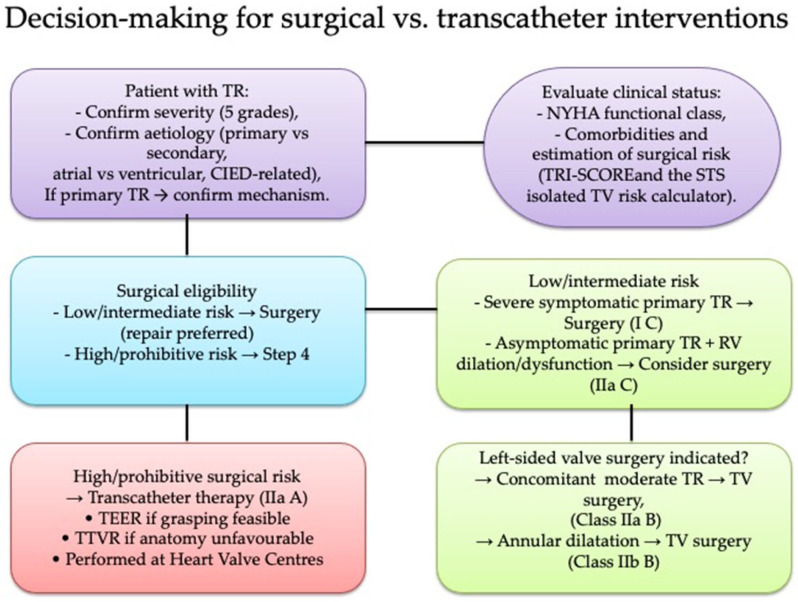
Management algorithm of tricuspid regurgitation: surgical vs. transcatheter approaches. Abbreviations: CIED: Cardiac Implantable electronic device, NYHA: New York Heart Association, RV: right ventricle, TEER: Transcatheter edge-to-edge repair, TR: tricuspid regurgitation, TTVR: trans-catheter tricuspid valve replacement, TV: tricuspid valve.

Primary TR remains an underrecognized condition with highly heterogeneous etiologies and limited dedicated evidence. This review contributes to filling important gaps by systematizing current knowledge on the diverse mechanisms underlying primary TR (Table 4); emphasizing its frequent underdiagnosis and its prognostic significance; and critically appraising the emerging role of transcatheter therapies in the absence of randomized trial data (Figure 5). By clarifying these aspects, we provide clinicians with a structured overview to support earlier recognition and individualized management, while also outlining priorities for future research to establish evidence-based treatment strategies.

## 5. Perspectives

While this review has summarized current knowledge and clarified key gaps in the understanding of primary TR, many questions remain unanswered. These open issues underscore the need for further investigation. Future research should focus on improving the understanding of the molecular and structural mechanisms underlying primary TR, as current knowledge remains limited compared with left-sided valvular disease. Large-scale prospective registries are needed to better define the natural history of the disease and to identify reliable imaging and biomarker-based tools for early diagnosis and risk stratification. In parallel, randomized controlled trials are essential to establish the role of transcatheter therapies, which currently rely mainly on observational data. From a clinical standpoint, earlier recognition of primary TR and timely referral to specialized heart valve centers will be key to improving outcomes. Integration of advanced multimodality imaging, RV functional assessment, and multidisciplinary decision-making should shape a more personalized approach, ultimately bridging the current gap between diagnosis and effective treatment.

## Figures and Tables

**Table 1:** This table represents the main differences between mitral valve prolapse and tricuspid valve prolapse.

Characteristics	Mitral Valve Prolapse	Tricuspid Valve Prolapse	References
Prevalence ^1^	Common (2–3% of the general population)	Very rare	[8,9,10]
Pathogenesis	Myxomatous degeneration with valvular interstitial cell activation, extracellular matrix disruption, leaflet thickening, and chordal elongation/rupture.	Poorly defined; may reflect the same myxomatous process as MVP or a distinct entity related to chordal/papillary abnormalities or RV remodeling.	[9,10,11]
Histopathology	Fibrosa degeneration, spongiosa expansion and myofibroblast cells activation	Unclear, hypothesized to share similar degenerative features	[11]
Imaging tools	Echo (2D/3D TEE), CMR widely validated	Challenging due to thin leaflets and variable anatomy; CMR increasingly used	[9,10]
Diagnostic criteria	Leaflet displacement >2 mm in parasternal long axis view	RA displacement > 2–3 mm (depending on leaflet) on echo or CMR	[9,10,11]
Surgical treatment	Established repair techniques	Annuloplasty, bicuspidalization, clover technique, Alfieri’s stitch	[13]
Transcatheter treatment	MitraClip widely used	TriClip^®^ used in primary TR; early data promising	[14,15]
Guideline indications	If severe and symptomatic MR related to MVP, class I indication for surgery	If severe and symptomatic TR, class I indication for surgery without RV dysfunction	[16,17]

^1^ Abbreviations: CMR: cardiac magnetic resonance, MR: mitral regurgitation, MVP: mitral valve prolapse, RV: right ventricle, TEE: trans-esophageal echocardiography, TR: tricuspid regurgitation.

**Table 2:** This table represents a short summary of the main features of tricuspid valve prolapse.

Tricuspid Valve Prolapse	Key Points	References
Prevalence ^1^	Very rare (<1%) in imaging studies, <50 cases described in the literature	[9,10]
Pathogenesis	Unclear; debated whether TVP is a distinct entity or part of the spectrum of myxomatous degeneration	[8,9,10,11]
Diagnostic criteria (echo- and CMR-based)	On TTE: atrial displacement of leaflet ≥ 2 mm (parasternal short-axis view),	[9,10]
Diagnostic challenges	On CMR: ≥3 mm displacement for septal leaflet, ≥2 mm for anterior/posterior leaflets.	[9,11]
Therapeutic options	Thin leaflets, anatomical variability, and rarity → underrecognition and misclassification as functional TR Echo (2D/3D TEE), CMR	
	-Surgical techniques: annuloplasty, Kay bicuspidalization, clover technique [13,16,17]	[13,14,15,16,17,18]
	-Transcatheter edge-to-edge repair (T-TEER, Triclip^®^) feasible in selected cases [14,15,18].	

^1^ Abbreviations: CMR: cardiac magnetic resonance, TEE: trans-esophageal echocardiogram, TTE: trans-thoracic echocardiogram, T-TEER: tricuspid edge-to-edge repair, TR: tricuspid regurgitation, TVP: tricuspid valve prolapse.

**Table 3:** This table represents diagnostic features, prevalence, and management of carcinoid heart disease with TR involvement.

Tricuspid Regurgitation Carcinoid Related	Key Points	References
Pathogenesis ^1^	Serotonin and vasoactive substances from neuroendocrine tumors bypass liver metabolism → right-sided valve fibrosis/retraction	[59,60,61]
Valve involvement	Primarily tricuspid and pulmonary valves; diffuse leaflet thickening, fibrosis, restricted mobility	[62,63]
Imaging features	Dilated annulus; fibrotic/retracted leaflets; 3D TTE improves assessment; Doppler: low gradient, dagger-shaped jet	[61,62,63,64,65]
Surgical therapy	TV replacement most common; operative mortality improved from 29% to ~5% in recent decades (Mayo Clinic series)	[66]
Transcatheter therapies	T-TEER often limited (leaflet retraction); TTVR may overcome anatomy, early reports promising	[67,68]

^1^ Abbreviations: TTE: trans-thoracic echocardiogram, T-TEER: tricuspid transcatheter edge-to-edge repair, TV: tricuspid valve, TTVR: transcatheter tricuspid valve replacement.

**Table 4:** Main features of primary tricuspid regurgitation.

Etiology	Mechanism of Regurgitation	Clinical Notes	References
Myxomatous degeneration/Flail leaflet	Redundancy, elongation or rupture of chordae tendineae	Probably associated with mitral valve prolapse	[11,12]
Traumatic/Iatrogenic	Chordal or leaflet rupture secondary to chest trauma, catheter injury and post EBM	Can present acutely with severe TR	[19,20,21]
Endocarditis	Leaflet destruction due to infection	Typically related to IV drug use or intracardiac devices or hemodialysis	[34,35,36,37,38,39,40,41]
Carcinoid heart disease	Fibrotic thickening and retraction of valve leaflets	Often involves tricuspid and pulmonary valves; right-sided endocardial fibrosis	[59,60,61,62,63]
Radiation induced	Fibrosis and calcification of the valve apparatus	Long latency; usually seen decades after chest radiotherapy	[78,79,80,81,82,83]
Congenital anomalies	Structural malformations of leaflets	Can be suspected in advanced age	[71,72]
Infiltrative diseases (e.g., amyloidosis, sarcoidosis)	Direct leaflet infiltration or annular involvement	May coexist with restrictive physiology or RV dysfunction	[89,90,91,92]

Abbreviations: EBM: endomyocardial biopsy, IV: intravenous, RV: right ventricle, TR: tricuspid regurgitation.

## Data Availability

The original data presented in the study can be found in the bibliographic references.

## Abbreviations

The following abbreviations are used in this manuscript:

AD	atrial displacement
AR	aortic regurgitation
AS	aortic stenosis
CA	cardiac amyloidosis
CHD	carcinoid heart disease
CMR	cardiac magnetic resonance
CS	cardiac sarcoidosis
EA	Ebstein’s anomaly
ECM	extracellular matrix
ECMO	extracorporeal membrane oxygenator
EMB	endomyocardial biopsy
ePTFE	expanded polytetrafluoroethylene
ESC	European Society of Cardiology
FDA	Food and Drug Administration
FDG	fluorodeoxyglucose
HIV	human immunodeficiency virus
IDUs	intravenous drug users
IE	infective endocarditis
LGE	late gadolinium enhancement
MMPs	metalloproteinases
MR	mitral regurgitation
MV	mitral valve
MVP	mitral valve prolapse
NTs	neuroendocrine tumors
PET	positron emission tomography
PR	pulmonic regurgitation
RA	right atrium
RV	right ventricle
RSIE	Right-sided infective endocarditis
RVGLS	RV global longitudinal strain,
TEE	transesophageal echocardiography
TTE	trans-thoracic echocardiography
T-TEER	tricuspid edge-to-edge repair
TR	tricuspid regurgitation
TTVR	transcatheter tricuspid valve replacement
TV	tricuspid valve
TVP	tricuspid valve prolapse
VHD	valvular heart disease
VICs	valvular interstitial cells
